# A novel composite hyaluronic acid/chitosan/polyvinyl alcohol ternary hydrogel scaffold for periodontal regeneration: an experimental study

**DOI:** 10.1186/s12903-026-09272-9

**Published:** 2026-07-20

**Authors:** Jaylane K. Ghonima, Mohy El-Din El-Rashidy, Gehan Kotry, Samir R. Nouh, Dina Nagui, Eman Thabet, Salma E. El-Habashy

**Affiliations:** 1 Department of Oral Medicine and Periodontology Oral Diagnosis and Oral Radiology, Faculty of Dentistry, Alexandria University, Champollion St, Alexandria, 21526 Egypt; 2 Department of Surgery, Faculty of Veterinary Medicine, Alexandria University, Alexandria, 21561 Egypt; 3 Department of Oral Biology, Faculty of Dentistry, Alexandria University, Alexandria, 21526 Egypt; 4 Department of Medical Physiology, Faculty of Medicine, Alexandria University, Alexandria, 21561 Egypt; 5 Center of Excellence for Research in Regenerative Medicine and Applications (CERRMA), Faculty of Medicine, Alexandria University, Alexandria, Egypt; 6 Department of Pharmaceutics, Faculty of Pharmacy, Alexandria University, Alexandria, 21521 Egypt

**Keywords:** Chitosan, Furcation Defects, Hyaluronic acid, Hydrogel, Periodontal Regeneration, Polyvinyl alcohol

## Abstract

**Background:**

Furcation defects are considered among the most challenging periodontal lesions to regenerate given the complexity of the periodontal apparatus, inaccessibility and the limited blood supply to the area. The aim of the current study was to create and characterize a novel ternary hydrogel composed of hyaluronic acid (HA), chitosan (CS) and polyvinyl alcohol (PVA) and to evaluate its potentials in periodontal regeneration of furcation defects in dogs.

**Materials and methods:**

A composite hydrogel scaffold composed of hyaluronic acid, chitosan and polyvinyl alcohol was successfully prepared and characterized in terms of SEM, swelling and mechanical behaviors, FTIR analysis as well as biocompatibility assay and direct cell-scaffold interactions. In an in vivo study, thirty-two critical size class II furcation defects were created in eight mongrel dogs and randomly allocated to group I, hydrogel group and group II, negative control group. Histological analysis and histomorphometric evaluation of percentage of newly formed bone area were used to evaluate the regenerative potential of the novel hydrogel after one and three months, postoperatively.

**Results:**

The prepared hydrogel was cytocompatible, had proper degradation rate, water uptake and mechanical strength as well as ease of handling clinically. Histologic results of the hydrogel group revealed superior bone, periodontal ligament and cementum formation compared to the negative control group at both time points. The hydrogel group showed a statistically significant increase in the percentage of newly formed bone surface area compared to the negative control group.

**Conclusions:**

The novel ternary hydrogel prepared using hyaluronic acid, chitosan and polyvinyl alcohol showed adequate cytocompatibility and mechanical properties. The in vivo results support that the novel scaffold might be effective for periodontal regeneration in furcation defects in dogs.

**Supplementary Information:**

The online version contains supplementary material available at 10.1186/s12903-026-09272-9.

## Background

The prime goal of conventional non-surgical periodontal therapy is to control microbial infection of the periodontium by eliminating bacterial biofilm, calculus, and bacterial toxins from the periodontally involved root surfaces. This is done using supra and subgingival scaling, root surface debridement and gingival curettage [1, 2]. Although these traditional measures give good clinical results, and help improve periodontal condition, their outcomes lack stability and they do not fully regenerate the lost tissues [3]. The ultimate goal of regenerating the lost tissues was then attempted through the concept of guided tissue regeneration (GTR). Using barrier membranes to prevent fast epithelial cells from penetrating the defect site, and give the slower osteoblasts the time needed to fill the defect with bone [4]. Several bone grafts, bone substitutes and other biomaterials were then used in conjunction with barrier membranes in an attempt to achieve complete periodontal regeneration [4, 5]. Despite the noticeable improvement of periodontal treatment outcomes using these techniques, their predictability and consistency remain questionable specially in cases with complex defect topography [6].

To maximize the odds for regeneration, the principle of tissue engineering was introduced in periodontal therapy [7]. It is based on filling the defect with a complex capable of stimulating the body’s regenerative process to properly regenerate the defective area. This complex should comprise a scaffold mimicking the extracellular matrix (ECM), stem cells and growth factors to allow the differentiation and proliferation of cells [8].

Ideal scaffolds for tissue engineering must be biocompatible, non-cytotoxic to maintain viability of cells, highly porous to allow cell attachment and proliferation as well as nutrient diffusion [9]. They also should have mechanical strength comparable to the target tissue and a degradation rate that matches the rate of new tissue formation [10]. Several polymeric materials have been used as scaffolds for regenerative medicine, tissue engineering, drug delivery and cell cultures [11].

Natural polymers have excellent biocompatibility and biodegradability as they resemble the composition of extracellular matrix which consists of polysaccharides, glycosaminoglycans, and various proteins [12]. However, their mechanical properties and biostability are low compared to the other synthetic group [12]. On the other hand, synthetic polymers can be tailored to have exceptional mechanical properties and stability excelling natural ones [13]. Yet, their biocompatibility and biodegradability are considered low compared to natural ones, they also lack inherent bioactivity [14].

Several polymers have been extensively investigated for periodontal regeneration. Chitosan (CS) is a natural polysaccharide that exhibits great biocompatibility, biodegradability, as well as intrinsic antimicrobial activity, in addition to hemostatic and adhesive properties [15]. In their review in 2020, Tao et al. [16], concluded that chitosan nanofibers can promote cell growth, osteogenic differentiation, angiogenesis of mineralized bone tissue, as well as facilitation of drug delivery in bone regenerative engineering [16].

Hyaluronic acid (HA) is a natural component of ECM and has a crucial role in tissue hydration. It is a biocompatible, bioactive, biodegradable, non-immunogenic and non-thrombogenic polymer with high water affinity [17]. Moreover, HA is a potent anti-inflammatory mediator, has an antioxidant effect and can activate different signaling pathways [18]. It also maintains morphologic organization by preserving extracellular spaces [15]. Hyaluronic acid has been used for periodontal therapy as an adjunct to non-surgical [19] and surgical [20] periodontal therapy showing favorable effects in both domains [19–21]. Additionally, HA scaffolds have bacteriostatic effects on certain bacterial strains including Aggregatibacter actinomycetecomitans (Aa) [22], which can be useful in periodontal regeneration. Moreover, studies have demonstrated that HA scaffolds have the ability to regenerate alveolar bone [23], and enhance the mineralization potential of osteoblasts [24].

Poly vinyl alcohol (PVA) is an FDA approved, water-soluble, synthetic polymer. Its biocompatibility, biodegradability and ease of synthesis facilitated its use in a wide range of applications including drug delivery, tissue regeneration and wound healing [25–27]. Delan et al. [28], in 2022 concluded that PVA nanofibers (NFs) showed histologic and radiographic signs of bone regeneration when placed in induced bone defects in rabbits. An additional vital advantage of using PVA as a scaffold for periodontal regeneration is its ability to inhibit epithelial ingrowth as proved by Zhou et al. [29], in 2020.

To combine the advantages of both natural and synthetic polymers, while minimizing their drawbacks, composite hydrogels are prepared by blending them together [30]. Hydrogels are cross-linked, biocompatible, readily available, three-dimensional, hydrophilic, porous, polymers that can be prepared in various controllable compositions [13, 31]. They have shown promising results when used as scaffolds for periodontal regeneration [32]. These hydrogels have a mesh-like structure that allows them to mimic the microenvironment of ECM facilitating cell attachment, proliferation and differentiation [11, 13].

Several combinations of polymers have been studied aiming to create the best scaffold for cell cultures, drug delivery and tissue engineering.

Chitosan and HA are frequently blended together in a hybrid scaffold for various applications including periodontal regeneration [33], cartilage repair [34], tissue engineering [35, 36], controlled drug delivery [37] and wound healing [38]. Similarly, chitosan is often mixed with PVA in a hydrogel which has several applications such as bone regeneration [39] and wound healing [26, 40]. Additionally, HA was also combined with PVA in a composite hydrogel that was evaluated for tissue engineering [41].

Given the proved potential of chitosan, hyaluronic acid and poly vinyl alcohol separately to enhance periodontal regeneration as before mentioned, and the belief that combining polymers together can further improve the outcomes. The aim of this study was to create a novel [42] ternary hydrogel composite comprising CS, HA and PVA and to characterize it in terms of scanning electron microscope (SEM), swelling and mechanical behaviors, Fourier transform infrared spectroscopy (FTIR) and to verify its biocompatibility. Secondarily, to perform a simple evaluation of the new hydrogel in periodontal regeneration in furcation defects in a canine model. This evaluation was carried out by histological evaluation and histomorphometric (quantitative) analysis of the percentage of newly formed bone area. The null hypothesis was that the newly created scaffold will not be beneficial for periodontal regeneration and will not result in new bone formation.

## Methods

### Materials

Hyaluronic acid (HA; CAS No. 9067-32-7, Advent ChemBio, India) and high molecular weight chitosan (CS; CAS No. 9012-76-4, Carl Roth GmbH, Germany) were used. Polyvinyl alcohol (PVA; CAS No. 9002-89-5, MW 14 kDa) was obtained from Adwic, El-Nasr Pharmaceutical Co. (Egypt). All other chemicals were of analytical grade.

### Preparation of innovative hyaluronic acid/chitosan/polyvinyl alcohol hydrogel scaffolds (HA/CS/PVA)

Hydrogel scaffolds were fabricated similar to a previous reports [43, 44]. Aqueous solutions of HA and PVA were prepared in deionized water, while CS was dissolved in 1% v/v acetic acid [45]. The innovative polymer blend included HA, CS and PVA at different final concentrations and HA/CS w/w ratios (6/1, 6/3, 6/6 and 3/3; Table 1). The prepared mixtures were then cast in glass molds and lyophilized (Lyoquest; Telstar, Spain). The novel freeze-dried hydrogel scaffolds (HA/CS/PVA) were sectioned into 3D blocks (5 mm × 3 mm × 2 mm) for further experiments.

**Table 1:** Optimization of the innovative hydrogel scaffolds using different polymer concentrations

Code	Polymer concentration (% w/v)		
	HA	CS	PVA
Hydrogel-1	6	1	2
Hydrogel-2		3	
Hydrogel-3		6	
Hydrogel-4	3	3	

### Characterization of the prepared hydrogel scaffolds

#### Scanning electron microscopy (SEM)

The ultrastructural morphology of the developed scaffolds was evaluated using SEM (JSM-IT200; JEOL, Japan). Sections were gold-coated for examination. Pore size distribution was recorded using image analysis (Fiji version 1.52p; National Institutes of Health, USA) of 30–50 fields [46].

#### Swelling behavior

The water uptake potential of the developed hydrogel scaffolds was determined, as previously reported [46]. Scaffolds were immersed in 2 mL phosphate-buffered saline (PBS) and allowed to swell at 37 °C and 50 rpm (Wisebath; Daihan Scientific Co. Ltd, South Korea). At different time points, scaffolds were blotted against filter paper and weighed (W_t_). Weights were then compared to scaffolds dry weights (W_0_). Percentage swelling was calculated using Eq. 1:

1 $$Swelling\:\left(\%\right)=\frac{{W}_{t}-{W}_{0}}{{W}_{0}}\times\:100$$

The maximum swollen weight (W_s_) was recorded and percentage loss in wet weight was calculated using Eq. 2 [46].

2 $$Loss\:in\:wet\:weight\:\left(\%\right)=\frac{{W}_{s}-{W}_{t}}{{W}_{s}}\times\:100$$

#### Mechanical behavior

The mechanical features of the developed hydrogel scaffolds were determined via texture analysis (CT3; Brookfield, USA). Scaffolds were wetted with 0.5 mL saline before analysis, then scaffolds were compressed at 1 mm/s to reach a target distance of 2 mm and trigger load of 0.05 N. The ultimate compressive strength values were recorded, and Young’s elastic modulus was determined from the stress-strain plots.

#### Fourier transform infrared spectroscopy (FTIR)

Functional group analysis was performed using Fourier transform infrared spectroscopy (FTIR; Agilent Cary 630; Agilent technologies, USA). Samples were scanned over 650–4000 cm^− 1^ at 2 cm^− 1^ resolution.

### Biocompatibility assay and direct cell–scaffold interactions of HA/CS/PVA scaffolds with BMSCs

First, a biocompatibility assay was done to assess the indirect effects of HA/CS/PVA released from each scaffold over a period of 2, 4 and 6 days on the cellular viability of BMSCs at 24 and 48 h by MTT assay with 4,5-dimethylthiazol-2-yl)-2,5-diphenyltetrazolium bromide solution using a previously established protocol [47].

Briefly, scaffolds were incubated with bone marrow stem cells (BMCS) complete medium consisting of low glucose Dulbecco’s modified Eagle’s medium (LG-DMEM, 1.0 g/L glucose; Lonza) supplemented with 10% fetal bovine serum (FBS, Biowest), 2mM L-glutamine, and 1% penicillin/streptomycin (P/S, 10,000 IU/mL/ 10,000 µg/mL, Lonza) in a 12-well plate at 37 °C with 5% CO_2_ for 2, 4, and 6 days (*n* = 5/each time interval). The conditioned media was collected at each time point. BMSCs were then seeded onto 96-well plates at a density of 5 × 10^3^ cells/well (*n* = 8 wells). Once confluent, the complete media was replaced with conditioned media exposed to scaffolds for 2, 4 or 6 days. Control wells were cultured in regular complete medium. BMCS were left in the scaffold conditioned media for 24 and 48 h before MTT assay was done [48].

For the MTT assay, the conditioned media was replaced with 100 µl of complete media containing MTT and incubated for 3 h at 37 °C with 5% CO_2_. Following incubation, media containing MTT was discarded and 100 µl of DMSO was added to dissolve formazan crystals. The plate was agitated for 10 min, and the absorbance at a wavelength of 570 nm was measured using an ELISA reader (Tecan, Infinite F50, Switzerland).

The cell viability of the controls was considered as 100% proliferation. Experiments were conducted using three independent biological replicates (*n* = 3).

To test for cell interaction on the surface of HA/CS/PVA scaffolds, the scaffolds were first sterilized by under UV lamp for 30 min. Following sterilization, 5 × 104 BMSC were seeded on each side of the scaffold in a well of a 24-well plate. The scaffolds with BMSC were incubated for 2 h (to allow for cell adhesion) then 0.5 ml complete media was added to the wells. The seeded scaffolds were incubated under standard conditions (37 °C, 5% CO₂) for 48 h to allow for cell growth. Following incubation, the scaffolds were washed two times with PBS and fixed with 4% PFA for 10 min. The scaffolds were then washed 3X with PBS before permeabilized with 0.1% TritonX for 15 min. F-Actin dye easy probes reagent (ThermoFisher; R37112) was applied to the permeabilized cells for 30 min (2 drops /ml media) and DAPI was added for 10 min in the dark. The scaffolds were mounted on cover slip and imaged using confocal imaging (Leica TCS SP5, Germany) and images were acquired using 20X objectives to confirm cell infiltration within the scaffold structure. This experiment was performed in duplicate [49].

### In vivo study

#### Aim of the experimental study

In the in vivo study we aimed to evaluate the regeneration potential of the novel hydrogel in surgically induced class II furcation defects in a canine model both histologically and histomorphometrically through assessing the percentage of newly formed bone surface area.

#### Study design and sample size

This was an experimental study conducted on a canine model. A total sample size of 32 defects in eight dogs was estimated using a 95% confidence level, and a power of 80%. Based on previous study, the mean % of bone area after 1 month was 9.77 ± 1.25% for negative control and 23.19 ± 7.94% for hydrogel group [50]. A comparison of means [51] using the higher standard deviation (7.94) to ensure sufficient power indicated a minimum of 7 sites per group per scarification interval to achieve an effect size of 1.8. This was increased to 8 sites per group to account for possible laboratory errors.

#### Inclusions and exclusions criteria

Eight healthy adult Mongrel dogs of 12–20 months old, weighing around 15 kg were included in the study.

Dogs who had previous surgeries, pregnant female and physically sick dogs were excluded from the study.

#### Randomisation and blinding

In each dog, four defects were prepared in mandibular premolars P3 and P4 bilaterally. The defects were randomly allocated to one of the two study groups: group I (hydrogel group) or group II (negative control group) using computer-generated simple randomisation software [52].

In this study, both the histological data assessor and the statistician were blinded to the study groups.

#### Animal participation and study approval

The animals were supplied and maintained by the animal house of Tissue engineering program, Faculty of Dentistry, Alexandria University, Egypt. The animal house granted full permission to conduct the current study. The study was approved by Animal Care Ethical Committee of Faculty of Dentistry, Alexandria University, Egypt (0735-07/2023) and it was performed following the instructions of Institutional Animal Care and Use Committee (IACUC) guidelines [53]. The study was conducted in accordance with ARRIVE (animal research reporting of in vivo experiments) guidelines [54].

#### Study setting

- Preparation of hyaluronic acid-integrated hydrogel was done at the department of Pharmaceutics, Faculty of Pharmacy, Alexandria University, Egypt.
- Surgical furcation defect creation and management were performed at the animal house of Tissue engineering program, Faculty of Dentistry, Alexandria University, Egypt. The dogs used in this study were kept under the same environmental conditions in the experimental animal house.
- Histological and histomorphometric evaluation were done in the department of Oral Biology, Faculty of Dentistry, Alexandria University, Egypt.

#### Surgical procedures

All surgical procedures were performed under the same general anesthesia regimen: sedation using intramuscular injection of Xylazine Hcl (1 mg/kg) (Xyla-Ject 20 mg/ml, Phoenix Pharm., Egypt), and induction with intramuscular injection of ketamine Hcl (10 mg/kg). Then, the anesthesia was maintained intravenously by xylazine (0.5 mg/kg) and ketamine (Hcl 3 mg/kg). After successful general anesthesia, local infiltration anesthesia was administrated using Articaine hydrochloride + 1:100,000 Epinephrine (Inibsa, Spain.) opposite to the mandibular premolars bilaterally.

**Fig. 1:**
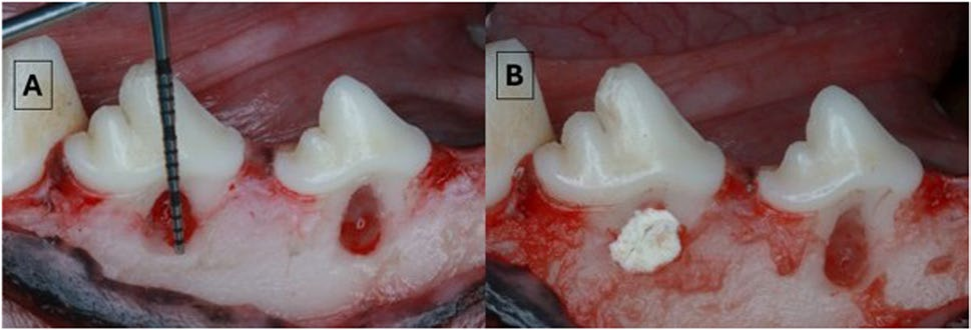
Surgically induced furcation defects and the management in both test and control groups (A&B). **A**: surgically created class II critical size furcation defects (5 mm vertically and 3 mm horizontally). **B**: hydrogel scaffold placement in P4 defect (test) and empty defect in P3 (control)

Then, mucoperiosteal flaps were reflected from second premolar to first molar areas. At P3 and P4 bilaterally, cortical plates were removed using small round burs, and induced critical sized grade II furcation defects of 5 mm height and 3 mm depth were created (Fig. 1A). Roots were scaled with periodontal curettes, then thoroughly washed with sterile saline. Reference notches were made on both mesial and distal roots at the new bone level to facilitate the orientation during histological analysis. The defects were randomly allocated to one of the two groups. Group I defects were filled with the novel hydrogel while Group II defects were left empty as negative control (Fig. 1B). Flaps were then repositioned and sutured with polypropolene sutures 2/0.

#### Postoperative care

Immediately postoperatively and for the following four days, dogs received intramuscular injections of ampicillin (300 mg/kg) and non-steroidal anti-inflammatory (0.04 mg/ kg body weight) (Declophen, Pharco Pharmaceuicals, Egypt). Sutures were removed after 10 days. Meanwhile, dogs were kept on soft diet to avoid trauma to the surgical sites.

#### Animal euthanization

Four dogs were euthanized using a lethal dose of anesthesia at 1 month time point while the rest were euthanized at 3 months time point. Then, mandibles were collected for analysis.

#### Histological and Histomorphometric analysis

Specimens were dissected, fixed in 10% neutral-buffered formalin for 2 days, washed, decalcified with 5% formic acid + Calcium acetate 45% for buffering, dehydrated with ascending concentrations of ethanol, cleared with xylene, and embedded in polymethylacrylate blocks. Sections were cut in a sagittal direction through the mesio-distal plane of premolars using a rotatory microtome and stained with Hematoxylin and Eosin (H&E) stain to be examined by light microscope for histological evaluation [55, 56].

The quantitative analysis (percentage of newly formed bone surface area) was analyzed from images taken under a light microscope at × 40 magnification using Image analysis software J 1.46 r software [57]. For each section three images were used and at least 10 sections were analyzed for each specimen. The region of interest (ROI) was identified by reference notches, and the metric calibration of the microscope was used as a standard demarcating an area of 3 × 4 mm. measurements were averaged and standard deviation was calculated [58].

### Statistical analysis

For the in vitro analysis, all experiments were run in triplicate.

For in-vivo analysis, normality of newly formed bone was checked using Shapiro Wilk test and Q-Q plots and normal distribution was confirmed; thus data was presented using mean and standard deviation (SD). Data were presented at animal level and analyzed using paired t test. All tests were two tailed and the significance level was set at p value < 0.05. Data was analyzed using IBM SPSS version 23 for windows, Armonk, NY, USA.

## Results

### Formulation of innovative (HA/CS/PVA)

In this study, polyelectrolyte complexation was adopted for the development of four different hydrogel scaffolds (Hydrogels 1–4), at different polymer concentrations and HA/CS w/w ratios (6/1, 6/3, 6/6 and 3/3; Table 1). Whereas PVA (2% w/v) was included for its positive impact of mechanical strength via freeze drying induced-physical crosslinking [43]. The fabricated scaffolds were characterized for different physicochemical features.

### Scanning electron microscopy (SEM)

**Fig. 2:**
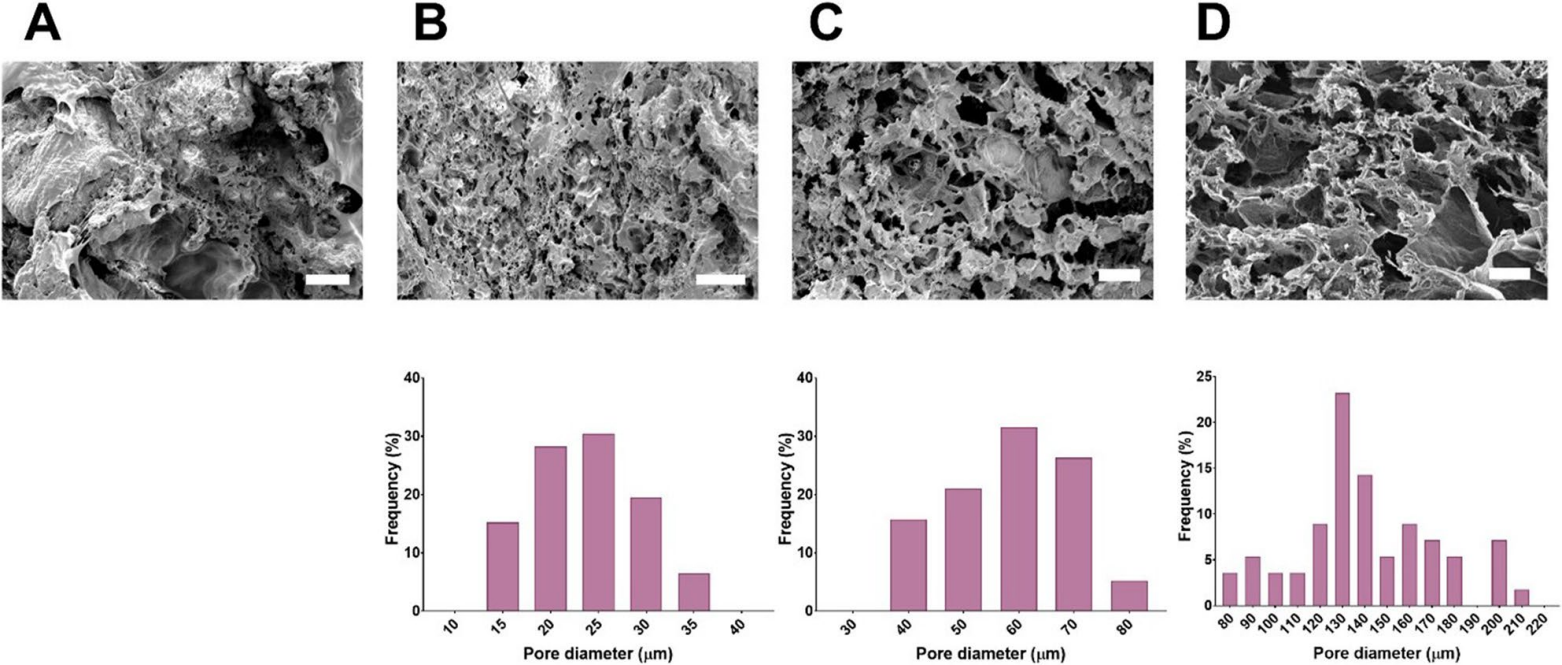
Ultrastructural morphology of the developed hydrogel scaffolds (A–D). Representative scanning electron micrographs (SEM; upper panel) and pore size distribution (lower panel) for Hydrogel-1 (**A**), Hydrogel-2 (**B**), Hydrogel-3 (**C**) and Hydrogel-4 (**D**). Scale bar = 100 μm. *n* = 30–50

The ultrastructural features and pore size analysis of all the developed hydrogel scaffolds (Hydrogels 1–4) were investigated. For all tested hydrogels, the obtained micrographs (upper panel; Fig. 2A–D) presented pronounced surface roughness, typical of freeze-dried scaffolds [45], which was less obvious for Hydrogel-1 (6/1 w/w HA/CS ratio). In fact, Hydrogel-1 presented a collapsed internal matrix with poor demarcation of pore structure (Fig. 2A). Increasing the CS content for the HA/CS ratios of Hydrogel-2 (6/3) and Hydrogel-3 (6/6) resulted in a well-defined, homogenous and interconnected pore structure, reflective of efficient polyelectrolyte complex formation and crosslinking of hydrogel matrix. Indeed, pore size analysis (lower panel; Fig. 2B and C) revealed a micron-sized porosity, with larger (*p* ≤ 0.05) pore size for Hydrogel-3 (57.35 ± 11.62 μm) than Hydrogel-2 (23.56 ± 5.8 μm), indicative of more effective complex formation at an equal HA/CS w/w ratio (6/6; for Hydrogel-3).

Interestingly, employing an equal HA/CS w/w ratio at low total polymer concentration (3/3; for Hydrogel-4) secured the significantly (*p* ≤ 0.05) highest pore size (142.12 ± 33.34 μm; Fig. 2D), a pore size range which is favorably optimum for osteoconduction [45].

### Water uptake potential

**Fig. 3:**
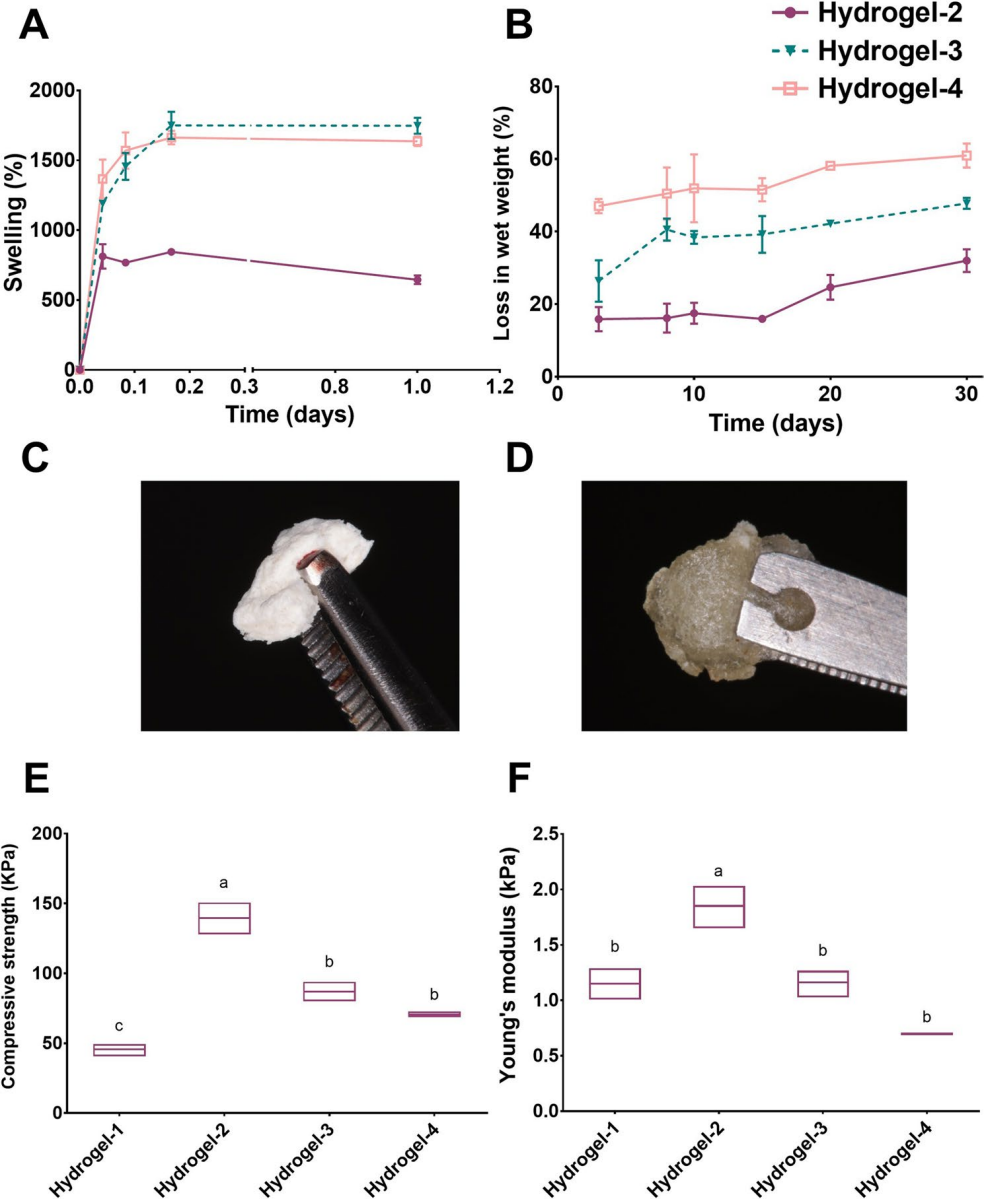
Characterization of the developed hydrogel scaffolds (A–F). Water uptake potential (**A**), loss in wet weight/erosion (**B**), ultimate compressive stress (**C**) and Young’s elastic modulus (**D**). Data represents SD, *n* = 3. Letters denote statistical significance: a > b > c, at *p* ≤ 0.05

In this work, the swelling behavior and loss in wet weight of hydrated scaffolds were monitored for 30 days. After incubation for 1 h, Hydrogel-1 exhibited complete liquefaction and erosion (data not shown). This behavior goes in line with the matrix collapse shown in the SEM micrographs (Sect.  3.2). For Hydrogels 2–4, swelling proceeded gradually over 24 h (1 day; Fig. 3A), where Hydrogel-2 presented the least percentage swelling (845 ± 11%) at 4 h compared to both Hydrogels-3 (1749.82 ± 97.77%) and Hydrogel-4 (1662.8 ± 48.6%). After 1 day, all hydrogel scaffolds exhibited notable erosion with different extents of wet weight loss that was controlled over 30 days (Fig. 3B). Specifically, the percentage loss in wet weight by 30 days increased following the pattern: Hydrogel-4 (60.94 ± 3.31%) > Hydrogel-3 (47.76 ± 1.5%) > Hydrogel-2 (31.99 ± 3.14%).

Macroscopic appearance of Hydrogel-4 is shown in (Fig. 3C&D), in its dry form (Fig. 3C) and 1 h after wetting (Fig. 3D).

### Compressive strength

Results of compressive strength indicated that Hydrogel-1 exhibited the lowest (*p* ≤ 0.05) compressive strength (45 ± 6.12 kPa; Fig. 3E). In contrast, Hydrogel-2 demonstrated a higher (*p* ≤ 0.05) mechanical strength (139.33 ± 16.02 kPa) than both Hydrogel-3 (87 ± 9.89 kPa) and Hydrogel-4 (70.66 ± 2.82 kPa), a pattern that closely accords with the pore size results (Sect.  3.2). A relatively similar pattern was obtained for Young’s modulus (Fig. 3F), where Hydrogel-2 presented the highest (*p* ≤ 0.05) modulus value (1.85 ± 0.19 kPa), indicating structural rigidity.

Taken together, Hydrogel-4 exhibited optimum physicochemical features for osteogenic application, with outstanding morphological features, optimum porosity, reasonable water uptake potential and compressive strength. It was therefore selected for further biological studies and henceforth denoted as HA/CS/PVA.

### Fourier Transform Infrared spectroscopy (FTIR)

Extensive functional group analysis was performed employing FTIR, comparing individual ingredients (HA, CS and PVA) to HA/CS/PVA.

**Fig. 4:**
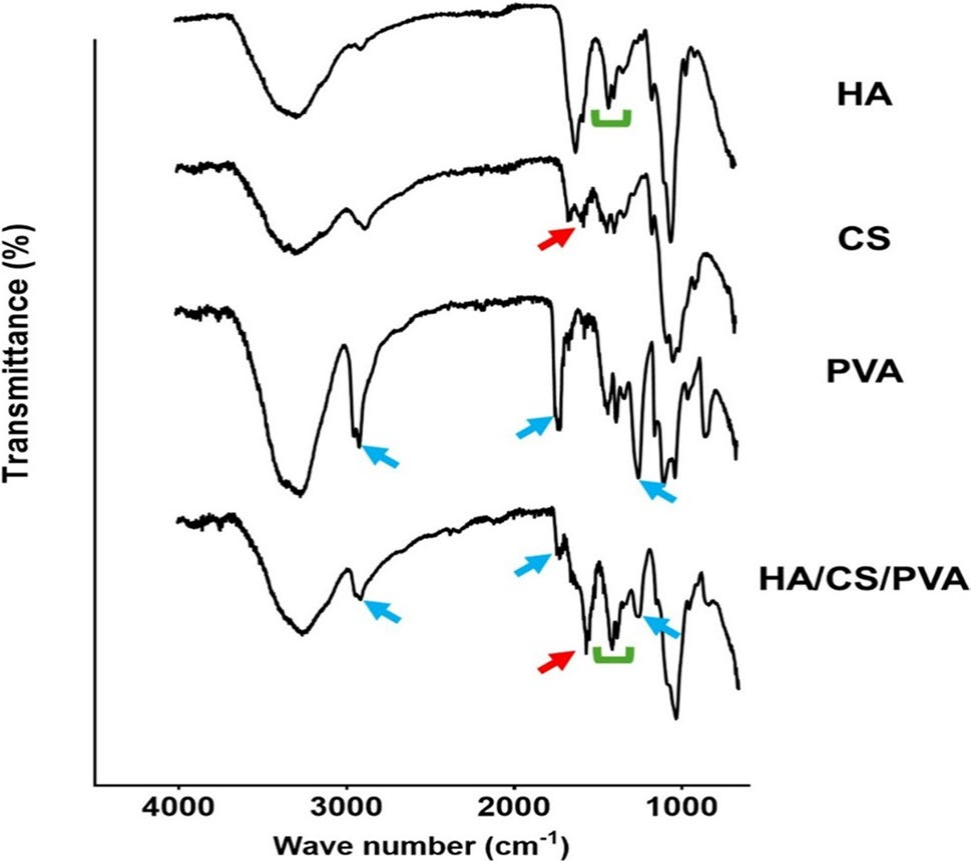
FTIR spectra of developed HA/CS/PVA scaffold in comparison to individual ingredients (HA, CS and PVA)

The FTIR spectrum for CS (Fig. 4) exhibits a broad band over 3000–3600 cm^− 1^ for the O-H and N-H stretching [46]. The bands at 2871, 1653 and 1564 cm^− 1^ correspond to -CH_2_ stretching [46], the C═O of amide band I [44] and the NH of amide band II [44], respectively. The spectrum for HA shows bands at 1610 and 1415 cm^− 1^ appointed to carboxylate group asymmetric and symmetric stretching, respectively. For PVA, the FTIR spectrum demonstrates peaks at 2913 and 1726 cm^− 1^ for the alkyl C-H and acetate C═C, respectively [43]. The FTIR spectrum for HA/CS/PVA exhibits all characteristic peaks of individual polymer components (Fig. 4). Interestingly, the sharp peak at 1563 cm^− 1^ corresponds to protonated NH^3+^ of CS, which interacts with the carboxylate of HA, shown at 1610 cm^− 1^, for polyelectrolyte complex formation [59].

### Biocompatibility and Direct Cell–Scaffold Interactions of HA/CS/PVA scaffolds with BMSCs results

**Fig. 5:**
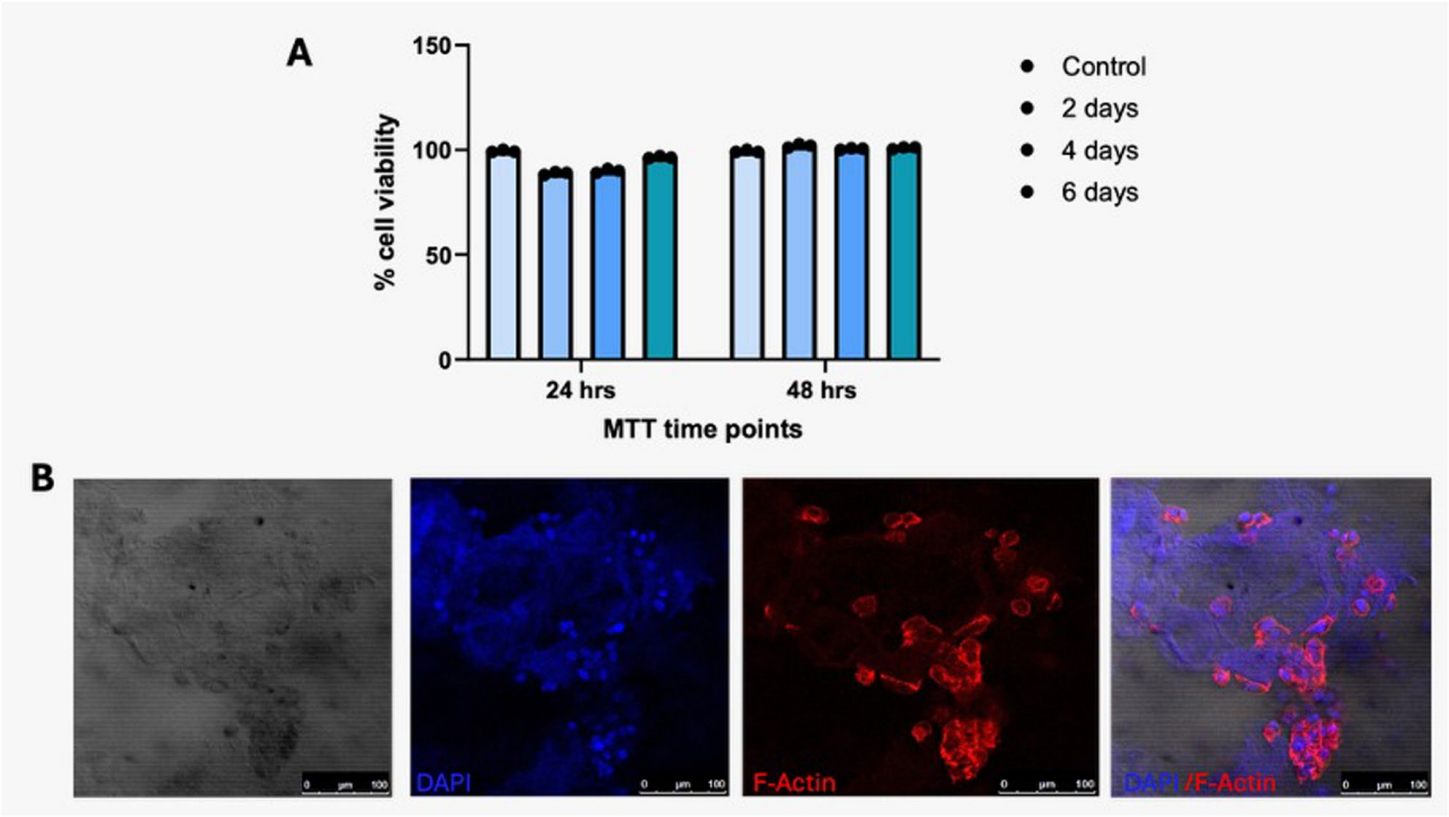
Biocompatibility and Direct Cell–Scaffold Interactions of HA/CS/PVA scaffolds with BMSCs. Cytotoxic effect of hyaluronic acid/chitosan/polyvinyl alcohol (HA/CS/PVA) scaffold material on BMSCs at 24 and 48 h (**A**). Cell viability is expressed as mean ± SD from three independent biological replicates (*n* = 3). Representative confocal microscopy images showing BMSCs cultured on the HA/CS/PVA scaffold (**B**). Cells were stained for F-actin (red) to visualize cytoskeletal organization and DAPI (blue) to label cell nuclei. Images demonstrate cell attachment, spreading on the scaffold surface. Scale bar: 100 μm

The cytotoxic effects of HA/CS/PVA hydrogel are exhibited in (Fig. 5A). The cytotoxic effect of the HA/CS/PVA scaffold on bone marrow–derived mesenchymal stem cells (BMSCs) was evaluated at 24- and 48-hours following exposure. Cell viability was assessed using quantitative in vitro assays, and all experiments were conducted with three independent biological replicates (*n* = 3). Data are presented as mean ± standard deviation (SD).

At 24 h, BMSCs cultured in HA/CS/PVA scaffold conditioned media (2 days soaking) exhibited high cell viability (88.8 ± 0.7) Although a slight reduction in viability as compared to the control group (99 ± 0.5), the overall cell survival remained above 85–90%, suggesting good cytocompatibility of the scaffold material at this time point. BMSCs cultured in conditioned media soaked for 4 days also showed high cell viability (89.9 ± 0.98).Similarly, BMSCs cultured with conditioned media soaked for 6 days showed high cell viability (96.4 ± 0.5).

At 48 h, BMSC viability remained stable compared with the 24-hour time point. Cell viability for BMSC cell cultured in all three conditioned media conditions (2, 4- and 6-days soaking) were 101 ± 0.8,100 ± 0.4, 100.9 ± 0.46% respectively). This indicates that prolonged exposure to the HA/CS/PVA scaffold did not induce delayed cytotoxic effects. (ns, where *p* > 0.05).

Images of the stained BMSs on scaffold show efficient adherence as shown by the F-actin staining which were widely dispersed along the surface of the scaffold (Fig. 5).

### In vivo study results

In this study, we investigated the in vivo periodontal regenerative potential of the innovative ternary hydrogel scaffold HA/CS/PVA. New bone formation was histologically and histomorphometrically analyzed at two time points (1 and 3-month post-operatively).

#### Histological results

**Fig. 6:**
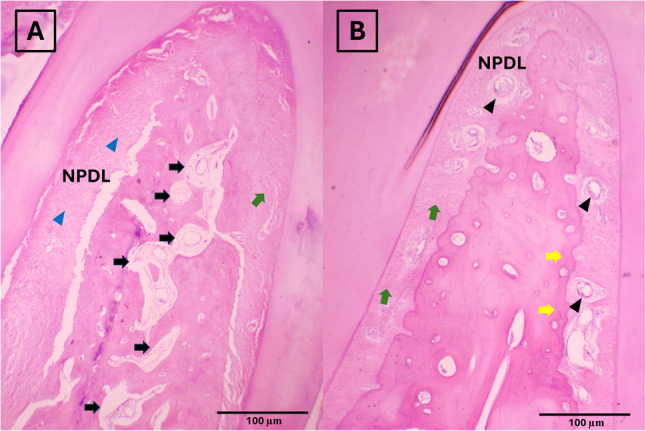
Hydrogel group light micrograph (L.M.) at 1 month (A) and 3 months (B) postoperatively. **A** L.M. of Hydrogel group 1 month postoperatively showing newly formed dense mature bone filling the defect. Large bone marrow cavities are still evident denoting in progress remodeling (black arrows). A continuous thick band of new periodontal ligament-like tissue (NPDL) (blue arrowhead) surrounds the newly formed bone. Most of the PDL fibers show poor orientation (blue arrowhead) with some well-oriented PDL fibers inserted into cementum-like tissue could be detected in some areas (green arrow). **B** L.M. of Hydrogel group 3 months postoperatively showing mature bone with small sized marrow spaces in the defect area. Normal width of PDL (NPDL) with properly oriented fibers inserted in cementum-like tissue (green arrow). Numerous interstitial spaces are detected (black arrowheads). Note remodeling lines within the newly formed bone denoting active maturation (yellow arrows). Note the hydrogel residues within a marrow space (black arrows)

**Fig. 7:**
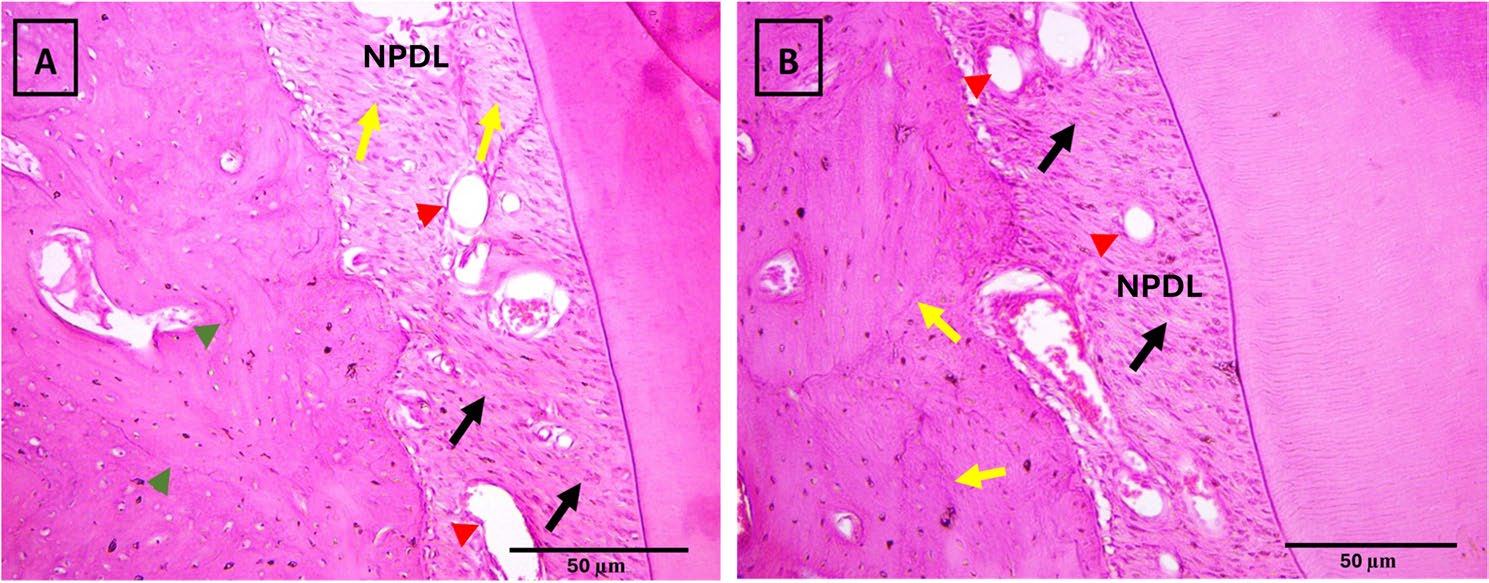
Showing high magnification of Hydrogel group at 1 month (A) and 3 months (B) postoperatively. **A** L.M. of Hydrogel group at 1 month follow up under 400x magnification showing group of fibers of new periodontal ligament -like tissue (NPDL) with typical dense oblique arrangement (black arrows), while other fibers are haphazardly arranged (yellow arrows). Newly formed mature bone appears filled with osteocytes with several darkly stained lines denoting on-going remodeling (green arrowheads). Note the medium and large-sized blood vessels within the interstitial spaces of NPDL (red arrowheads). **B** L.M. of Hydrogel group 1 month showing normal width of new PDL (NPDL) with properly oriented oblique fibers (black arrows). Most of blood vessels in NPDL appear smaller in size (red arrowheads). Note the new dense bone with typical concentrically- arranged osteons denoting maturation (yellow arrows)

After one month, the histologic examination of hydrogel group specimens showed a dense newly formed bone filling up to the fornix. Large bone marrow spaces were present within the bone denoting ongoing bone maturation. A wide continuous band of newly formed PDL-like tissue with mostly poorly organized fibers was found to surround all the new bone. Some well-oriented PDL fibers were inserted into the cementum-like tissue alongside the root surface. (Figs. 6A and  7A).

By three months postoperatively, the newly formed bone became mature showing small marrow spaces. Numerous remodeling lines were seen within the newly formed bone representing active maturation of the bone. The PDL-like tissue showed normal width with properly oriented fibers inserted into the cementum-like tissue. Several interstitial spaces were noted within the PDL-like tissue. Persistent hydrogel residues did not fully degrade and were seen in some marrow spaces. (Figs. 6B and  7B).

**Fig. 8:**
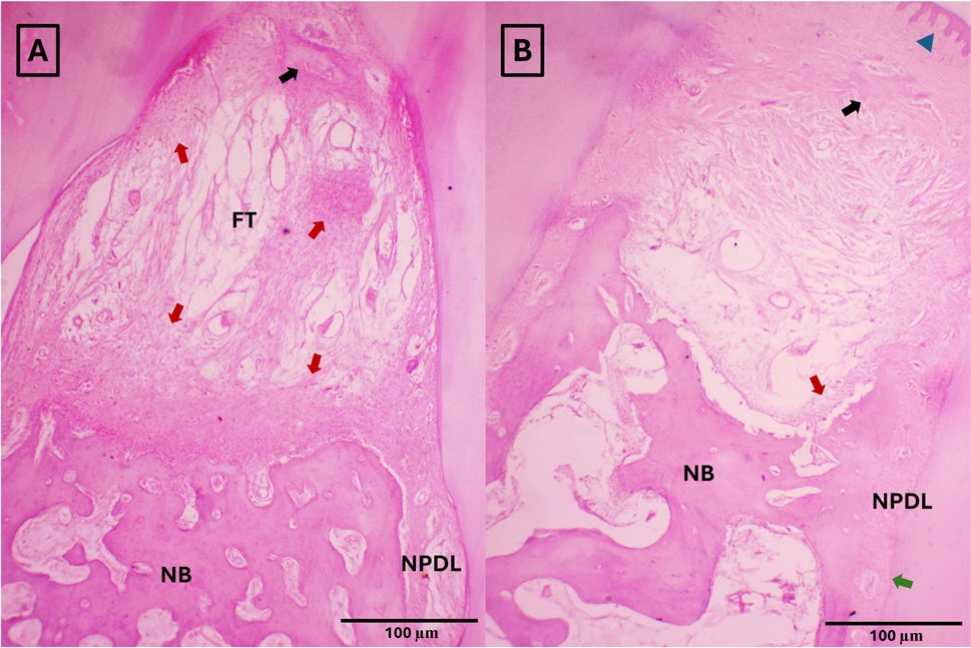
Negative control group light micrograph (L.M) at 1 month (A) and 3 months (B) postoperatively. **A** The L.M. of negative control group at 1 month postoperatively showing new bone formation at the apical part of the defect with numerous marrow spaces (NB). A new periodontal ligament-like tissue with irregular orientation of fibers is seen adjacent to the newly formed bone apically (NPDL). The rest of the defect is filled with dense fibrous tissue (FT) with increased inflammatory cells infiltration (red arrows). Note newly formed bone spicules within the fibrous tissue at the summit of the defect (black arrow). **B** The L.M. of negative control group at 3 months postoperatively showing bone formation at the apical part of the defect (NB) but with wide marrow spaces. The PDL-like tissue shows improved orientation of fibers (NPDL). New cementum-like tissue can be observed between new PDL-like tissue and root surface (green arrow). Most of the defect is still filled with fibrous tissue (FT) infiltrated by some inflammatory cells (red arrow). Sporadic new bone spicules exist near the summit of the defect (black arrow). Epithelial infiltration at the summit of the defect can be detected (blue arrowhead)

**Fig. 9:**
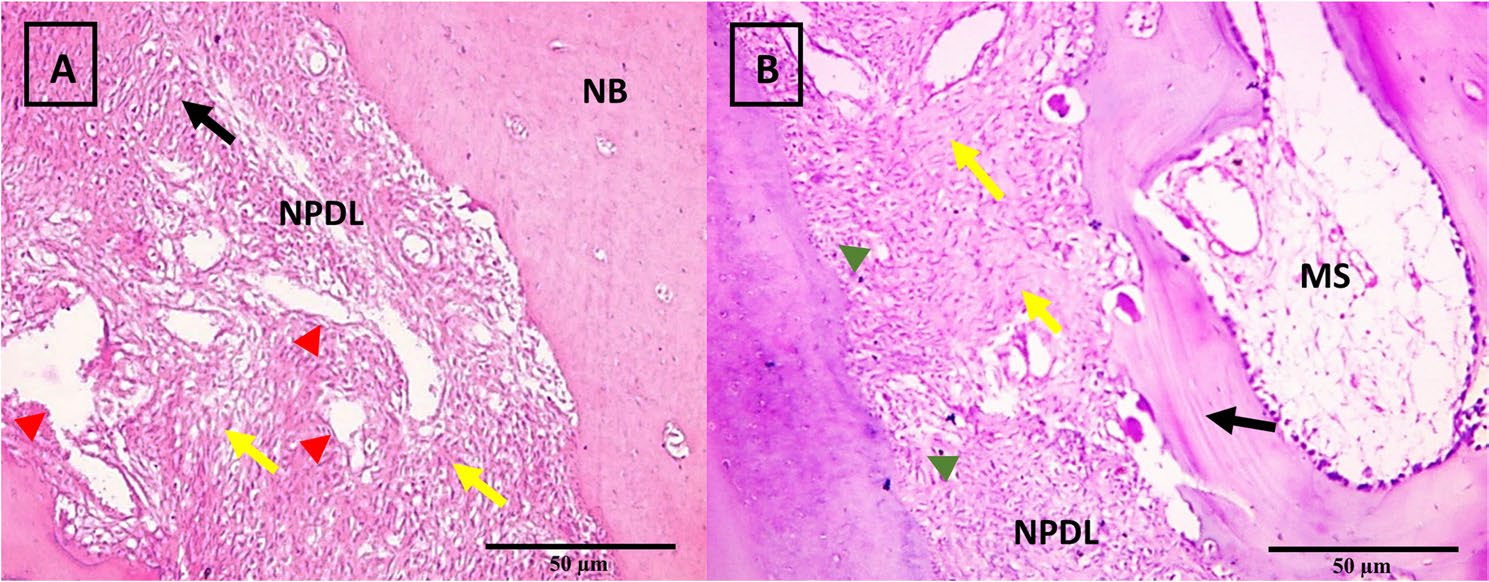
Showing high magnification of negative control group at 1 month (A) and 3 months (B) postoperatively. **A** The L.M. of negative control group at 1 month showing newly formed bone with numerous small marrow spaces (NB). A developing periodontal ligament-like tissue with primary orientation of fibers can be noticed (black arrow). However, most of the defect is filled with dense fibrous tissue (FT) with increased inflammatory cells infiltration (yellow arrows). Note the large-sized blood vessels occupying large areas of the defect (red arrowheads). **B** The L.M. of negative control group at 3 months showing mature bone formation denoted by incremental lines (black arrow). However, large-sized marrow space is detected (MS). Reduced width of new PDL-like tissue (NPDL) is noticed with dense fibers showing improved orientation along the bone side (yellow arrows). While, towards the new cementum-like tissue, the NPDL fibers are still irregularly arranged (green arrowheads)

One month postoperatively, the histologic evaluation of the negative control group revealed some new bone formation at the apical part of the furcation defects while the rest of the defect till the fornix was filled with fibrous tissue (FT). The newly formed bone contained numerous fibrovascular marrow spaces and a heavy population of osteocytes. An isolated new bone spicule was noted near the summit of the defect within the fibrous tissue. Heavy inflammatory cell infiltration was observed throughout the FT. A new periodontal ligament-like tissue with poorly oriented fibers and wide interstitial spaces was observed adjacent to the new formed bone. (Fig. 8A and  9A).

At three months postoperatively, the histologic examination of negative control group showed slightly more bone deposition with fewer osteocytes population. The marrow spaces were still wide, but the PDL fibers were properly arranged and inserted in the cementum-like tissue. The rest of the defect was filled with fibrous tissue (FT) with inflammatory cells infiltrates. Epithelial cells infiltration was observed in some specimens (Figs. 8B and  9B).

#### Histomorphometric results

Measuring the newly formed bone surface area was performed by the same trained examiner, and the intraclass calibration coefficient was 0.88. At the 1-month time point, the novel hydrogel group showed a significantly high (P value < 0.001) percentage of newly formed bone (36.02 ± 4.42%) compared to the negative control group (3.34 ± 0.70%). By 3 months, the hydrogel group showed further improvement in the percentage of newly formed bone (41.80 ± 3.24%) opposed by only (4.28 ± 0.53%) in the control group. (Table 2)

**Table 2:** Comparison of the percentage of newly formed bone area between negative control and hydrogel after 1 month and 3 months

		Negative control	Hydrogel	*Mean difference (95% CI)*	*P* value
		Mean ± SD			
*Animal level*	1 month	3.34 ± 0.70	36.02 ± 4.42	32.68 (26.47, 38.90)	< 0.001*
	3 months	4.28 ± 0.53	41.80 ± 3.24	37.52 (32.62, 42.42)	< 0.001*

*Statistically significant difference at *p* value < 0.05, *P* value: Paired t test

## Discussion

In the current study, we successfully developed and characterized a novel ternary hydrogel composed of hyaluronic acid (HA; 3% w/v), chitosan (CS; 3% w/v), and polyvinyl alcohol (PVA; 2% w/v), aiming at providing the perfect scaffold for periodontal regeneration. Incorporating both natural and synthetic polymers was intentional to enhance bioactivity, biocompatibility, and mechanical strength, which are the three critical attributes of a functional tissue engineering scaffold.

These specific polymers were selected in this study for their documented bone regenerative potential [28, 40, 60]. Hyaluronic acid is a major constituent of natural ECM, which explains its excellent biocompatibility [22]. Additionally, HA has other physicochemical and biological properties that make it uniquely suitable for tissue engineering. It is viscoelastic, bacteriostatic, antibacterial, anti-inflammatory, antiedematous [22, 61] and when crosslinked, it shows improved mechanical properties needed for regeneration [24]. Pilloni et al. in 2021 [62] proved that HA is effective in reducing pocket depth and improving clinical attachment level in humans when used for treating intrabony defects in conjunction with single flap approach [62]. Moreover, in experimental studies conducted on dogs, cross-linked HA was proven to be effective in inducing periodontal tissue regeneration in the treatment of recession [63], two-walled osseous defects [64] and furcation defects [60].

Several in vitro studies proved the ability of chitosan to promote neovascularization, osteoblastic differentiation and proliferation, and enhance the healing process through its antimicrobial and anti-inflammatory properties [65–67]. Therefore, it has been widely used in the regeneration of different tissues, including cartilage, bone, and skin [16, 68]. Moreover, in a clinical trial, Meenakshi et al., in 2021 [69] used chitosan nanohydrogel in the management of intrabony defects in chronic periodontitis patients and proved its bone regenerative potential [69].

Being natural polymers, both HA and CS show great biocompatibility and biodegradability, however they both lack mechanical strength and have rapid degradation rates in their pure forms [70]. This necessitated adding a synthetic polymer to improve their mechanical properties. Polyvinyl alcohol PVA was added to serve this purpose in addition to its advantages in tissue regeneration [71]. Indeed, freeze drying induced-physical crosslinking of PVA has been reported to positively impact scaffold mechanical strength [43]. Studies have shown that PVA has good tissue repair potentials due to its porosity, ability to swell and viscoelastic properties [72, 73]. In 2021, Xie et al. [74], successfully fabricated and characterized a collagen/hydroxyapatite/polyvinyl alcohol hydrogel for cartilage repair. The resultant scaffold could effectively repair cartilage defects [74]. Moreover, Huang et al. [75], in 2023 conducted an in vitro evaluation of a flexible film of CS/PVA to be used as a membrane barrier for periodontal regeneration. The authors demonstrated that by increasing the percentage of PVA, the resultant hydrogel showed less porous structure and lower degradation rate [75].

In this work, we rationalized that polyelectrolyte complexation between CS and HA would result in an effectively crosslinked hydrogel scaffold matrix [46, 76], which would be structurally favorable for the proposed osteogenic functionality. In this regard, the effect of different polymer concentrations on scaffold physicochemical features for the optimization of scaffold composition were thoroughly investigated.

Scaffolds for osteogenic regeneration should exhibit favorable surface topography and structural porosity for successful osteogenic regeneration [77]. Specifically, surface roughness provides anchorage sites for cell attachment and regeneration, whereas interconnected porosity offers the required space for cell recruitment, migration and proliferation as well as nutrient diffusion and fluid exchange [43]. Therefore, microscopic features of the developed scaffolds were monitored in relation to different scaffold compositions (Hydrogels 1–4). Poor polyelectrolyte complex formation might have contributed to the collapsed matrix at the low CS content for Hydrogel-1 (6/1 w/w HA/CS ratio), with subsequent matrix collapse during sample processing [59]. Whereas higher CS content was associated with more efficient polyelectrolyte complexation and well-defined pore formation (Hydrogels 2–4). Complex formation might have afforded more polymer junction points, resulting in larger pore size upon freeze drying [45]. Nath et al. [59] reported an increase in pore size of HA/CS polyelectrolyte complex hydrogel compared to plain CS scaffolds. Whereas the lower total polymer concentration for Hydrogel-4 might have allowed for higher void volume to be occupied by ice crystals during freeze drying.

Scaffolds for osteogenic functionality should properly take up physiological fluids, swell and act as a cell-friendly microenvironment that is able to sustain hosted cells for an optimum period of time, sufficient for bone ingrowth [45]. For this purpose, monitoring swelling and weight loss potential are essential parameters. The obtained water uptake patterns could be closely related to pore size range shown in the SEM micrographs (Sect. 3.2). In particular, the low pore size of Hydrogel-2 might be associated with low fluid diffusion and hence diminished matrix swelling and erosion extent. On the other hand, it is possible that the higher pore size of Hydrogel-4 than Hydrogel-3 contributed more fluid diffusion, water uptake by the polymer matrix and hence higher swelling and erosion.

For successful osteoconduction, the employed scaffold should possess reasonable mechanical strength as well as structural elasticity to sustain a microenvironment with osteogenic cell cues fostering effective bone growth [43]. In this regard, the compressive strength and Young’s modulus were recorded for the fabricated hydrogel scaffolds as indicators for mechanical strength and elasticity, respectively. Compressive strength results further accorded with the pore size pattern, where an increase in mechanical strength was associated with lower pore size. It is expected that poor complexation and structural collapse of Hydrogel-1 (SEM results in Sect. 3.2), can be associated with poor scaffold mechanical strength [46]. It has been previously reported that structural porosity and pore size are inversely related to mechanical strength [78]. The lower Young’s modulus value for Hydrogel-4 (0.96 ± 0.01 kPa) verifies high structural elasticity and resilience, favorable for cell interaction as well as effective bone tissue integration [77]. Physicochemical optimization verified that Hydrogel-4 possessed optimum physicochemical features favorable for the proposed osteogenic functionality. Specifically, Hydrogel-4 presented optimum surface topography, pore size range for osteoconduction, water uptake potential and erosion/weight loss pattern. Moreover, Hydrogel-4 exhibited reasonable compressive strength and elasticity. Indeed, FTIR examination verified the establishment of CS/HA polyelectrolyte complexation for the effective fabrication of HA/CS/PVA.

Biocompatibility is a prerequisite for any hydrogel for biomedical applications [79]. In the present study, all conditioned media exposed to HA/CS/PVA scaffolds for 2,4 and 6 days maintained BMSCs viable in the MTT assay. These results were comparable to the complete media used as control with no statistically significant difference, which denotes the great biocompatibility of HA/CS/PVA hydrogel.

To the best of the authors’ knowledge, the present study is the first to histologically evaluate the periodontal regenerative potential of a HA/CS/PVA ternary hydrogel in grade II furcation defects in a canine model. This specific animal model was selected in our study based on its similarity to humans regarding periodontal anatomy, disease progression, clinical outcomes of applied procedures, as well as its availability and simple handling [80]. Critical size furcation defects are incapable of spontaneous healing, thus represent a suitable method to evaluate the regenerative potential of tested biomaterials [50]. Moreover, this model has been successfully used in other studies to assess the use of different biomaterials for periodontal regeneration, and hence is considered as a useful and valid model [81, 82].

Periodontal regeneration of furcation defects poses a challenge not only due to the complexity of the periodontal tissue anatomy, but also due to the presence of complex microbial biofilm. This microbial burden hinders healing and can prevent cell differentiation, proliferation and attachment. Therefore, using a scaffold with antimicrobial properties may be beneficial in periodontal regeneration in such defects. Interestingly, our prepared hydrogel possesses a bacteriostatic property owed to the presence of HA which has been proven to inhibit bacterial contamination in both surgical and non-surgical periodontal therapy [83, 84].

In the current study, at one month follow up, histological examination of the hydrogel group revealed a newly formed bone filling all the defect until the fornix with large number of osteocytes and wide fibrovascular marrow spaces indicating active bone formation. It also showed a thin continuous layer of cementum-like tissue immediately adjacent to root surfaces and a thick continuous layer of PDL-like tissue surrounding the newly formed bone. Whereas in the control group, at the one month follow up the new bone formation was limited to the base of the defects and some isolated islands of bone trabeculae within the marked granulation tissue that filled all the defect. Periodontal attachment formation was rarely demonstrated and when present, it showed poorly arranged fibers that were limited to the apical parts of the defects. Additionally, marked inflammatory cell infiltration was observed in the negative control group specimens. Moreover, in some negative control group specimens epithelial growth was observed in the defects near the fornix.

The findings of our study group were superior to those of Momose et al. [85], who studied the effect of collagen and fibroblast growth factor-2 composite hydrogel in furcation class II defects in dogs. In their study, after four weeks the group treated with collagen hydrogel alone showed new bone formation limited to the apical part of the defect that did not fill it up till the fornix. Similarly, our results were also better than those of Abdelrasoul el al [50], in their experimental study, where they evaluated injectable bioactive melatonin-loaded alginate/chitosan/ β-TCP hydrogel in class II furcation defects in dogs. In that study, histological evaluation after four weeks revealed that the furcation defects treated with only hydrogel showed slight bone formation with disorganized PDL fibers as well as epithelial infiltration into the defect preventing proper periodontal regeneration.

By the three months follow up, the test group showed further bone maturation, with smaller marrow spaces and fewer trapped osteocytes, as well as improvement in the PDL-like fibers orientation. Some hydrogel residues were visible in the marrow spaces indicating the controlled degradation rate of the scaffold leading to persistent stimulation of bone formation for up to three months duration. The presence of residual hydrogel particles three months postoperatively can be explained by using a synthetic polymer in the novel hydrogel resulting in delaying the degradation of the scaffold [86].

On the other hand, in the present study the negative control group at three months postoperatively showed minimal bone formation and maturation. Additionally, epithelial growth was monitored in some specimens of the negative control group at three months. These results are in accordance with several experimental studies that showed limited or no healing in the negative control group [50, 60, 82].

The histomorphometric (quantitative) results evaluating the percentage of newly formed bone showed significant new bone formation with the novel HA/CS/PVA hydrogel reaching 35.33 ± 4.19% after one month and increasing to 42.40 ± 2.76% by three months. Opposed by only a minimal percentage of newly formed bone of 3.30 ± 0.74% and 4.27 ± 0.63% in the control group in one and three months, respectively. The significantly higher percentage of newly formed bone in test group can be explained by the ability of individual CS, HA and PVA to promote bone regeneration as evidenced in several in vitro studies [86–88], and this potential was amplified due to combining them together. In a review article, Tian et al. [89], in 2022 concluded that CS can stimulate osteoblasts proliferation by increasing mineralization and calcium binding genes expression resulting in improved bone repair. Similarly, HA was found to enhance osteoblast activity, improve collagen type I production and increase TGF-β1 secretion, therefore improving bone formation [88]. According to Hu et al. [86], PVA can boost cell adhesion, upregulate osteogenic gene expression in osteoblasts and initiate mineralized matrix deposition.

Similar to our study, several experimental studies [60, 90, 91] evaluating different forms and compositions of hydrogels in furcation defects proved the superiority of their scaffolds over negative control groups in periodontal regeneration. This might be attributed to the three-dimensional, porous nature of hydrogels which facilitates cell migration, adhesion, and proliferation, as well as invasion of microvasculature, thereby enhancing bone regeneration [65].

As beforementioned the superior results in our study are due to the synergistic bone regenerative effect resulting from combining CS, HA and PVA.

Since this study focused on the preparation and characterization of the ternary hydrogel, several limitations involving the evaluation of periodontal regeneration were encountered and should be considered when interpreting the findings. First, the absence of radiographic analysis of the defects that could give an overview of the newly formed bone volume and density. Second, our histological analysis was limited to Hematoxylin and Eosin (H&E) staining, which provides fundamental morphological information but may not capture the full complexity of tissue regeneration like other special stains. Another limitation of the current study is using an animal model, although the canine model closely resembles human periodontal anatomy and healing, extrapolation of the findings to human clinical situations should be performed cautiously. Finally, histomorphometric analysis focused on a single parameter, namely the percentage of newly formed bone surface area, which may offer a restricted view of the overall healing response.

Future studies are needed to provide a more comprehensive understanding of the regenerative potential and the underlying mechanisms of action of the novel hydrogel. They should incorporate larger sample sizes, radiographic assessment, a wider array of staining mechanisms, and the measurement of multiple relevant parameters. In addition to evaluating the hydrogel in other forms of periodontal defects and loading it with different therapeutic materials. Furthermore, evaluating this novel hydrogel in tissue engineering with cell and cell-free therapies are highly recommended given the properties of the created scaffold that were proven by the characterization and the in vivo study. Finally, comparisons with established regenerative therapies are necessary before clinical translation can be considered.

## Conclusion

Within the limitations of this study, the novel ternary hydrogel prepared using HA, CS and PVA demonstrated cytocompatibility, degradation rate compatible with periodontal tissue regeneration, appropriate water uptake and mechanical strength, as well as clinically acceptable handling characteristics. Histological and histomorphometric findings of the in vivo study suggested that the developed scaffold may support periodontal tissue regeneration and enhance new bone formation compared with untreated defects.

## Supplementary Information

Supplementary Material 1.

## Data Availability

The datasets used and /or analyzed during the current study are available from the corresponding author upon reasonable request.

## Abbreviations

HA: Hyaluronic acid
CS: Chitosan
PVA: Polyvinyl alcohol
GTR: Guided tissue regeneration
ECM: Extracellular matrix
SEM: Scanning Electron Microscope
PBS: Phosphate-buffered saline
FTIR: Fourier transform infrared spectroscopy
BMSCs: Bone marrow stem cells
MTT: 4,5-dimethylthiazol-2-yl)-2,5-diphenyltetrazolium bromide solution
FBS: Fetal bovine serum
ELISA: The enzyme-linked immunosorbent assay
